# SDM-Assist software to design site-directed mutagenesis primers introducing “silent” restriction sites

**DOI:** 10.1186/1471-2105-14-105

**Published:** 2013-03-22

**Authors:** Abhijit Karnik, Rucha Karnik, Christopher Grefen

**Affiliations:** 1 Department of Computer Science, University of Bristol, Bristol, UK; 2 Molecular, Cell & Systems Biology, University of Glasgow, Glasgow, UK

**Keywords:** Site-directed mutagenesis, SDM, PCR, Cloning, Restriction endonucleases, Oligonucleotides, Software

## Abstract

**Background:**

Over the past decades site-directed mutagenesis (SDM) has become an indispensable tool for biological structure-function studies. In principle, SDM uses modified primer pairs in a PCR reaction to introduce a mutation in a cDNA insert. *Dpn*I digestion of the reaction mixture is used to eliminate template copies before amplification in *E. coli*; however, this process is inefficient resulting in un-mutated clones which can only be distinguished from mutant clones by sequencing.

**Results:**

We have developed a program – ‘SDM-Assist’ which creates SDM primers adding a specific identifier: through additional silent mutations a restriction site is included or a previous one removed which allows for highly efficient identification of ‘mutated clones’ by a simple restriction digest.

**Conclusions:**

The direct identification of SDM clones will save time and money for researchers. SDM-Assist also scores the primers based on factors such as Tm, GC content and secondary structure allowing for simplified selection of optimal primer pairs.

## Background

The ever-increasing quality of DNA proof-reading polymerases has facilitated numerous PCR-based approaches for mutating wild type DNA sequences. Several different approaches for site-directed mutagenesis (SDM) have been suggested and successfully established (for review, see [1]). One of the fastest and most reliable approaches is the QuickChange™ (Stratagene, La Jolla, USA) method in which two overlapping oligonucleotides are directly used in a PCR reaction using a plasmid-based gene as template [2,3]. Simple transformation of the resulting PCR product in *E. coli* allows isolation of the mutated DNA. Prior to transformation in *E. coli*, digestion with *Dpn*I (NEB, Hitchin, UK), an enzyme that degrades methylated DNA, is needed to reduce the amount of wild type template. However, this digestion is often incomplete and traces of template result in recovery of wild type plasmids that can only be distinguished from mutant DNA through sequencing, a time- and cost-intensive process. In addition, hemi-methylated DNA deriving from chimeric plasmid formation of mutated and wildtype single strand DNA is hardly digested through *Dpn*I under normal circumstances [4].

Despite the common notion that *Dpn*I digestion is sufficient to eliminate the wild type plasmid DNA, an evaluation of all mutated clones generated through SDM in the past five years in our lab (more than 100 different constructs) revealed that a rescue of 20-30% of un-mutated, wildtype plasmids after *Dpn*I digest is inevitable. To circumvent this problem researchers have chosen to additionally introduce a restriction site through silent mutations in the DNA sequence [5-10]. This unique identifier allows fast and reliable determination of mutated clones facilitating pre-selection of clones to be sent for sequence verification; however, designing primers manually to such an extent is cumbersome and time-consuming. Given that SDM primers are long, it is critical to have the best possible thermodynamic characteristics (such as GC content, melting temperature (Tm), etc.) to ensure that they would work successfully in an SDM reaction.

There is web-based software available to design SDM primers; there is also software available that scores designed primers on the basis of their thermodynamic characteristics to help the researcher choosing the best match. However, there is no single programme that incorporates both these functions in a user friendly way and in addition allows introduction of a unique identifier.

## Implementation

### SDM-Assist overview

The program is implemented in Adobe AS3 language which compiles into an Adobe AIR package. The main advantage of this choice is the cross-platform compatibility of Adobe AIR. Thus, the application can leverage the platform’s native application programming interfaces (APIs) for reading file/clipboard contents as well as writing and opening the results with the platform’s default file editors. It also allows us to provide the user with an intuitive graphical user interface (GUI) which supports drag and drop functionality for most actions. The program allows the user to generate all possible combinations of sequences containing the desired mutations. In a further step the user can then narrow down to a single sequence that is used as the basis for the downstream primer creation. At the same time the program lists all physical properties of the DNA sequence that are relevant for optimal primer design such as melting temperature, number of nucleotides exchanged, GC content and more. The researcher thus has access to all the details required to select a desirable sequence without having to transfer the results to another application for further processing.

### Algorithm

The algorithm consists of multiple steps. Each step is optimized to allow the generation of all the results very quickly (optimally less than a second for up to 50 bp sequences with multiple mutations and existing RE sites). In contrast, the same process carried out by hand would take much longer and is potentially prone to human error. The optimizations are at various steps of the process as follows:

a) Introducing a mutation:

The program provides the flexibility of specifying just the amino acid required for mutation. Since the genetic code bears some level of redundancy (e.g. serine can be represented by six different codons), the program generates all possible candidate sequences in the first selection step. In case of more than one mutation being introduced, the candidate list contains all possible permutations using all possible codons for all mutations. (e.g. two serine mutations will result in thirty-six candidates).

b) Restriction site search and replace:

The program searches for existing restriction sites and assigns replacements with the least number of nucleotide changes. This is carried out in two steps: For searching the restriction sites, the program uses the native regex search functionality of AS3 language. To eliminate the restriction site, the program identifies the codons over which the restriction site is spread and then performs a best match replacement which takes into account the number of nucleotide changes and also the GC content (for equal number of changes) to arrive at the best replacement. The program also optimizes removal of overlapping restriction sites by going for the least number of changes that eliminate both restriction sites.

c) Insertion of restriction sites:

The last step of the algorithm inserts restriction sites to provide a unique identifier for mutagenised clones. The program uses a reverse sliding-window approach for the insertion. While all prior actions are performed on a nucleotide-based string, this operation requires a conversion back to amino acid-based strings. The palindromic restriction site sequence is converted into multiple amino acid sequences. For example a six nucleotide restriction site which is not in-frame can be represented as NN/NNN/N where N can be A, T, C or G. The total number of sequences that can contain the restriction site is sixty-four. These sixty-four sequences can code to different amino acid sequences. More combinations arise when the sliding window moves the restriction site start away from the open reading frame. The algorithm then searches for insertion locations by matching the generated amino acid sequences within segments of the candidate sequence. For each match found, the algorithm inserts the specific restriction site at the exact location as nucleotides, thus keeping the original amino acid sequence intact. By converting nucleotide sequences back into amino acid sequences, the search is simplified and shortened to smaller strings and thus speeding up the search process.

d) Scoring:

The scoring step assigns scores to all generated primers as an additional level of filter criteria for the user. Seven parameters are included for the scoring and are assigned different weights. These parameters are GC content (2x weight), nucleotide changes (3x weight) and melting temperature (Tm), 3’-GC content, 3’- and 5’-ΔG and repeats (1x weight). Each parameter accounts for 100 points at its optimal value which are a Tm between 66-74°C, an overall GC content between 40-60%, a 3’-GC content of 50%, an unstable 3’-end (3’-ΔG values of -4.74 to -5.62 kCal/mol), a stable 5’-end (5’-ΔG values of -13.50 to -12.62 kCal/mol), no repeats and a minimum of 2 nucleotide changes. In theory, the weighted score can result in a maximum score of 1000 points and would equal 100% in the scoring column.

## Results and discussion

We have developed a standalone program that designs and evaluates SDM primers within seconds. Primer design is based on an optimised strategy using partially overlapping primer pairs which increases the likelihood of primer-template annealing over primer self-annealing, hence, significantly enhancing the outcome of the SDM [11-13]. The main feature of the program, however, is the introduction of new or removal of previous restriction sites thereby adding a unique identifier to each mutation. This is useful to distinguish between wild type and mutated sequence in general but it also allows differentiating between different point mutations of the same wild type gene in particular. The age of recombinational cloning allows researchers to maintain their genes-of-interest in cloning vectors (“Entry vectors”) from which, through simple one-step recombination reaction, the gene can be transferred in any other expression vector [14]. Creating many different point mutants of the same gene may increase the risk of contaminations through human error in subsequent cloning reactions. Recombined clones derived from sequence-verified Entry clones do not need another round of sequence verification since no further PCR amplification is needed, hence a unique restriction site specific for each mutation would serve as a simple and reliable identifier.

To our knowledge, there are only three oligonucleotide design programs that allow introduction of a “silent” restriction site during SDM primer design. SILMUT [15] was designed in 1992 and The Primer Generator [16] appeared in 1999. We could not find a working link for SILMUT which was intended as a web-based application, while The Primer Generator, designed as a standalone executable, is limited to a DOS-based text-entry intensive interface lacking any of the features of a modern GUI. Therefore we were unable to compare the performance of these programs to SDM-Assist. In 2005, however, a Java applet called SiteFind was published [5]. This web-based tool allows the input of just about 400 nucleotides of DNA, albeit not via copy and paste functionality. The user is presented with all three potential reading frames to choose from and via double-clicks on the desired amino acid can replace it with the desired mutation. The next screen allows exclusion/addition of restriction sites to include in the screening procedure and on the final page the nucleotide sequence is given including the restriction site that was introduced. Compared to SiteFind, SDM-Assist can handle a variety of methods of selecting the input sequence (copy&paste, drag&drop, etc.). It can easily handle inputs up to 40,000 nucleotides. Hence, SDM-Assist overcomes the limitation of SiteFind that prevents the user of entering a whole gene thereby reducing the length of the tool-chain (where the researcher uses different software to perform different steps of the oligonucleotide design process). Additionally, SDM-Assist also allows the manipulation and mutation of the input sequence in a user-friendly format and helps in designing the primers and calculation of their thermodynamic parameters. SiteFind apparently lacks these features and its output is limited to merely suggesting a potential mutation in a sequence, not presenting the actual oligonucleotide.

It is possible to scan a primer to incorporate “silent” restriction sites using an online tool called Watcut (http://watcut.uwaterloo.ca/). The programme available on the Website of the University of Waterloo identifies restriction sites created by any number of mutations using all commercially available type II restriction enzymes and calculates the Tm for the primer. However, it does not give the option to silently mutate out an existing restriction site nor is it a one-stop shop for SDM primer design.

**Figure 1:**
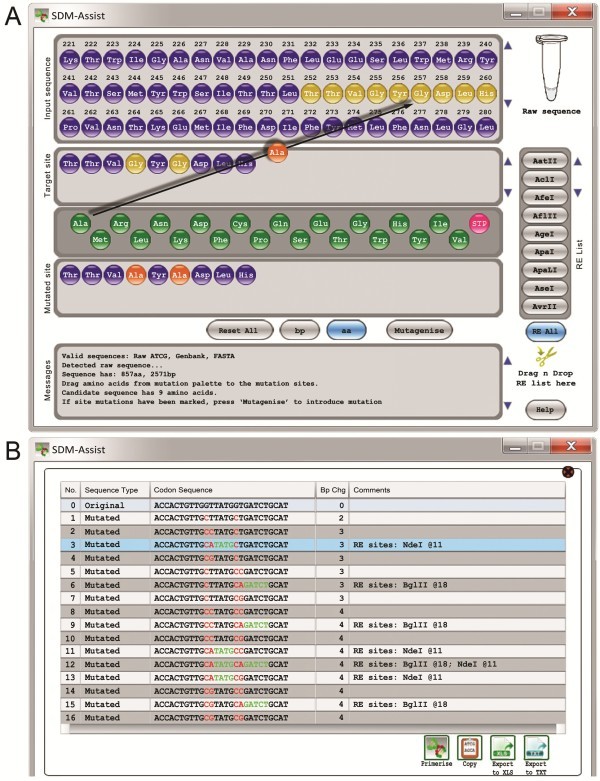
**Screenshots of SDM-Assist while creating primers to mutate the *Arabidopsis *potassium channel AKT1.** (**A**) Dragging and dropping of Alanine onto the Glycine residues at position 255 and 257 (black arrow) of the AKT1 sequence creates a stretch of 9 amino acids (below, “Mutated site”) that includes Alanine (orange) instead of Glycine (yellow) residues. Pressing the “Mutagenise” button opens a new window (**B**) that includes all possible nucleotide sequences derived from those 9 amino acids. The table highlights the mutated nucleotides in red and resulting palindromes which create a restriction site in green. The user has to choose one of the mutated sequences and press the button “primerise” to get a list of potential primers (see Table 1).

SDM-Assist, however, overcomes the drawbacks of existing software. Once the DNA sequence is pasted (See Figure 1A) it automatically gets translated into an amino acid sequence including a ruler for easier identification of target sites. Simple drag and drop allows choosing which amino acid is to be replaced with which (See Figure 1A, black arrow). In addition, right-click on any amino acid switches through the different codons of that particular amino acid. Choosing this option forces the program to use the desired codon when including the mutation (in case a certain codon bias is desired). It should be noted that additional mutations, even silent ones, might influence expression efficiency of the protein depending on the organism or system used. For further convenience the researcher can choose which restriction enzymes should be used by simply selecting from a list (See Figure 1A, right). It is also possible to add a user-defined list of restriction enzymes by dragging and dropping of a text-based file that contains the name of the restriction enzyme followed by its recognition sequence (tab or space delimited). Clicking the ‘mutagenise’ button opens a new window that gives a pre-evaluation of the sequence around the mutation(s) (See Figure 1B). It also highlights potential restriction sites that were present or new ones that can be introduced. The researcher is required to choose one of the sequences from which potential primer pairs will be calculated (See Figure 1B, line 3, highlighted in blue). Clicking the ‘primerise’ button will open a detailed list of possible oligonucleotides based on the pre-selected sequence (Table 1). This list can be saved as Excel or tab-separated text file by pressing either of the two corresponding buttons (See Figure 1B, bottom). This also resets the program and a new round of primer design can be started.

**Table 1:** Primer output sheet

**No.**	**Orientation**	**Primer (5**^′^**-3**^′^**)**	**Score [%]**	**nt changes**	**Tm [°C]**	**nt**	**GC%**	**3**^′^**ΔG**	**5**^′^**ΔG**	**Runs**	**Rep.**	**RE sites**	**3**^′^**GC%**	**Comments**
1	Forward	ACCACTGTTGCATATGCTGATCTGCATCC	69	3	72.16	29	48.28	−8.07	−7.71	0	0	1	50	RE sites: *Nde*I @11
2	Forward	ACCACTGTTGCATATGCTGATCTGCATCCT	70	3	72.75	30	46.67	−7.72	−7.71	0	0	1	50	RE sites: *Nde*I @11
3	Forward	ACCACTGTTGCATATGCTGATCTGCATCCTG	69	3	74.64	31	48.39	−8.2	−7.71	0	0	1	75	RE sites: *Nde*I @11
4	Forward	ACCACTGTTGCATATGCTGATCTGCATCCTGT	64	3	75.06	32	46.88	−7.96	−7.71	0	0	1	50	RE sites: *Nde*I @11
5	Forward	ACCACTGTTGCATATGCTGATCTGCATCCTGTG	66	3	76.82	33	48.48	−6.85	−7.71	0	0	1	50	RE sites: *Nde*I @11
22	Reverse	GCAGATCAGCATATGCAACAGTGGTTAGAGTAG	73	3	70.8	33	45.45	−5.5	−8.27	0	0	1	50	RE sites: *Nde*I @10
23	Reverse	TGCAGATCAGCATATGCAACAGTGGTTAGAGTAG	73	3	72.58	34	44.12	−5.5	−8.65	0	0	1	50	RE sites: *Nde*I @11
24	Reverse	ATGCAGATCAGCATATGCAACAGTGGTTAGAGTAG	73	3	72.64	35	42.86	−5.5	−8.52	0	0	1	50	RE sites: *Nde*I @12
25	Reverse	GCAGATCAGCATATGCAACAGTGGTTAGAGTA	68	3	70.2	32	43.75	−5.48	−8.27	0	0	1	25	RE sites: *Nde*I @10
26	Reverse	TGCAGATCAGCATATGCAACAGTGGTTAGAGTA	68	3	72.04	33	42.42	−5.48	−8.65	0	0	1	25	RE sites: *Nde*I @11

Only the first five Forward and Reverse primers are shown. The program offers a total of 21 Forward and 21 Reverse primers. The output table contains the physical properties of calculated oligonucleotides which are used as a basis for the scoring algorithm. (see Text for details).

Evaluation of primers is based on a positive scoring system taking physical properties (Tm, GC content, 5’/3’ stability, repeats [17,18]) as well as methodical requirements (palindromes, mismatches) of the oligonucleotides into account. Physical primer properties have been carefully revised adding a maximum of one hundred points for ideal values in each category and stepwise deduction of points for less optimal values. To counterbalance for methodical requirements of SDM where primer-template interaction is pivotal, GC content is weighed twice as strong and nucleotide mismatches three times as strong as other variables. A total of one thousand points (=100%) are possible for the perfect primer. For example, as SDM is based on mismatching primers which naturally would devalue a potential score, nucleotide mismatches of just one or two do not lead to a deduction of points. Three to four or five to six mismatches lead to a deduction of fifty or eighty points, respectively. More than seven mismatches are penalized with zero points and devalue a primer significantly (30% in total) as this category is weighed three times. This, however, guarantees that a primer with too many mismatches, which would undoubtedly lead to failure in a subsequent SDM reaction, is neglected by the researcher because of its weak score. The scoring system thus assists in selecting a primer pair that is more likely to give a successful SDM reaction.

**Figure 2:**
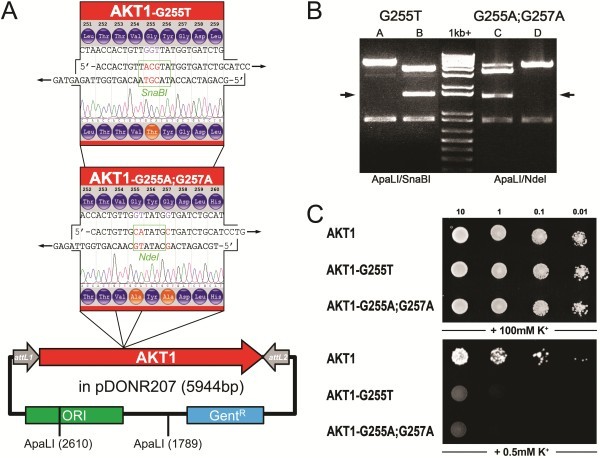
**Mutating the pore domain of the *Arabidopsis *potassium channel AKT1 and functional consequences *in vivo*.** (**A**) Wild type AKT1 in the Gateway® Entry vector pDONR207 is used as template for SDM of the GYG-motif within the pore-domain of the voltage-gated potassium channel AKT1 which is critical for formation of the channel-pore. SDM-Assist suggested primers for a single mutation of the Glycine 255 to Threonine adding a *SnaB*I-site, and for mutating both Glycines to Alanine introducing an *Nde*I site within the gene. Both restriction sites were absent in the wild type sequence. (**B**) DNA restriction analysis of randomly picked colonies after SDM (for PCR conditions, see Material and methods) demonstrates that distinction between wild type (lane 1 and 4) and mutated sequences (lane 2 and 3) becomes easily possible through the addition of a restriction site: an additional band appears in mutated plasmids (arrows). Sequence verification (trace file) is incorporated in (**A**). (**C**) Functional consequence of the single or double mutation: The yeast strain SGY1528 [22] which is disrupted in potassium uptake can only grow on high K^+^-medium (+100 mM) or on low K^+^-medium (0.5 mM) if an exogenous K^+^ transporter (such as wild type AKT1) is expressed. Disruption of the GYG-motif in the mutant AKT1-G255T and G255A;G257A renders the channel dysfunctional on low K^+^-medium, the yeast fails to grow. Equal amounts of yeast were dropped as dilution series on CSM-Leu^-^, His^-^, Trp^-^ media with final potassium concentrations of 100 mM or 0.5 mM. Growth on plates with high K^+^-concentration was monitored after 2 days and on low K^+^-plates after 8 days at 30°C. AKT1 and mutants were expressed using the yeast vector pMetYC-Dest [21].

To test the functionality of the program and the feasibility of a “silent” restriction site, we have mutated the *Arabidopsis thaliana* potassium channel AKT1 [19]. The GYG signature sequence in the pore domain of potassium channels is vital for ion permeability [20]. We have tested the program through addition of two different mutations in this region: (i) a single mutation, Glycine 255 to Threonine (G255T) and (ii) a double mutation of both Glycines to Alanine (G255A; G257A). Primers with the highest score were chosen for SDM and yielded successfully mutated clones (See Figure 2A and B). These were analysed through restriction digest and sequence verification (GATC Biotech AG, Konstanz, Germany). Correctly mutated constructs were recombined in yeast expression vectors [21] and transformed in the K^+^-deficient yeast strain SGY1528 [22]. Growth under K^+^-limiting conditions demonstrated that the introduced mutations successfully rendered the channels dysfunctional (See Figure 2C).

The objective of SDM-Assist is to simplify, shorten and error-proof the process of generating SDM primers while giving the researcher the ability to compare and choose between multiple alternative primers in an efficient manner. SDM-Assist also helps shorten the tool-chain used to select the primers. SDM-Assist fully supports drag and drop functionality and is well suited for touch-screen based devices. For the future, we envision a service based approach where the final selected sequence could be directly sent to the oligonucleotide supplier of choice thus enabling a seamless experience. Additional desired features comprise the ability to customize the scoring algorithm, save customised restriction site lists and user preferences. However, as of now, the absence of these features doesn’t hinder SDM-Assist from performing its primary objective to design functional SDM oligonucleotides including unique identifiers.

## Conclusions

There is an abundance of primer design software available some of which even allow the design of SDM primers. However, to our knowledge, SDM-Assist is the first stand-alone primer software that features the inclusion of a “silent” restriction site and a scoring algorithm for output primers. The software is compatible for both major operating systems Windows (XP and higher) and MacOS (10.6 and higher); primers are evaluated, scored and provided alongside their physical parameters in an output Excel or text-based file. With its overall intuitive and up-to-date design SDM-Assist will help researchers to save time and money when designing primers and performing SDM.

## Material and methods

### Plasmids, oligonucleotides and strains

As template for the SDM reactions we used the construct pDONR207-AKT1 [6]. The oligonucleotides to mutate this plasmid were designed using SDM-Assist. For the single site mutation of the Glycine residue 255 to Threonine the sequences 5’-ACCACTGTTGCATATGCTGATCTGCATCC and 5’-GCAGATCAGCATATGCAACAGTGGTTAGAGTAG (see Table 1, No. 1 & 22) were chosen. For the double mutation of the Glycine residues 255 and 257 to Alanine we used 5’-CACTGTTGCATATGCTGATCTGCATCCTG and 5’-TGCAGATCAGCATATGCAACAGTGGTTAGAG, respectively. Plasmids were recovered and amplified in *E. coli* strain Top10 (Life Technologies, Paisley, UK). For the yeast complementation assay mutated and wildtype AKT1 in pDONR207 was recombined using LR clonase II (Life Technologies, Paisley, UK) into yeast expression vector pMetYC-Dest [21].

### PCR conditions and recovery of plasmids

The SDM reaction was set up in two individual reactions one for each primer (0.3 μM final concentration). The reactions were set up in KOD Hotstart buffer and a final concentration of 0.2 mM each dNTP, 2.25 mM Mg^2+^, 5% DMSO, 20 ng template DNA and one unit of KOD Hotstart Polymerase (Merck, Darmstadt, Germany). An initial 95°C denaturation for two minutes was followed by cycles of 30 seconds denaturation at 95°C, one minute annealing at 55°C and synthesis of six minutes (one minute per kbp) at 70°C. After twelve cycles the two individual reactions were pooled and subjected to another 15 cycles of 30 seconds of denaturation at 95°C and seven minutes of synthesis at 70°C omitting an individual annealing step to increase specificity of primer template binding. The PCR reaction was followed by an overnight digestion at 37°C through addition of 2 μl (4000 units) of *Dpn*I (NEB, Hitchin, UK). 5 μl of the PCR reaction were subsequently transformed in *E. coli* strain Top10. Ten colonies per plate were isolated and analysed via restriction digest and sequencing (GATC Biotech, Konstanz, Germany).

### Yeast complementation assay

The yeast strain SGY1528 [22] was transformed using the Lithium acetate/PEG/ssDNA method as described previously [23]. A pool of 5-10 transformed colonies was grown overnight at 30°C in complete supplement media containing 100 mM K^+^, but lacking Leucine, Tryptophan and Histidine (CSM-L^-^,W^-^,H^-^). The optical density of cells was adjusted to an OD_600_ of ten. 5 μl of yeast cells in a decade-decreasing dilution series of OD_600_ 10, 1, 0.1 and 0.01 were spotted on solid CSM-L^-^,W^-^,H^-^ media with the addition of either 100 mM K^+^ (growth control) or 0.5 mM K^+^. Growth of yeast colonies was monitored after one week of incubation at 30°C.

## Availability and requirements

**Project name:** SDMAssist

**Project home page:**http://www.psrg.org.uk/sdm-assist.html

**Operating system(s):** Platform independent (Tested for Mac OS 10.6, Windows XP and above)

**Programming language:** ActionScript AS3

**Other requirements:** Adobe AIR 2 or higher (http://get.adobe.com/air/)

**License:** Non-commercial use only. [CC BY-NC-ND]

**Any restrictions to use by non-academics:** Contact authors for permission.

## Competing interests

The authors declare that they have no competing interests.

## Authors’ contributions

AK, RK and CG conceived and assembled the ideas for the program, AK programmed the software, CG performed experiments and wrote the manuscript with input from AK and RK. All authors read and approved the final manuscript.

## References

[B1] Ling MM Robinson BH Approaches to DNA mutagenesis: an overview Anal Biochem 1997 254 157 178 10.1006/abio.1997.2428 9417773

[B2] Braman J Papworth C Greener A Site-directed mutagenesis using double-stranded plasmid DNA templates Methods Mol Biol 1996 57 31 44 8849992 10.1385/0-89603-332-5:31

[B3] Wang W Malcolm BA Two-stage PCR protocol allowing introduction of multiple mutations, deletions and insertions using QuikChange site-directed mutagenesis Biotechniques 1999 26 680 682 10343905 10.2144/99264st03

[B4] Lu L Patel H Bissler JJ Optimizing DpnI digestion conditions to detect replicated DNA Biotechniques 2002 33 316 318 12188183 10.2144/02332st03

[B5] Evans PM Liu C SiteFind: a software tool for introducing a restriction site as a marker for successful site-directed mutagenesis BMC Mol Biol 2005 6 22 10.1186/1471-2199-6-22 16321147 PMC1314889

[B6] Grefen C Chen Z Honsbein A Donald N Hills A Blatt MR A novel motif essential for SNARE interaction with the K(+) channel KC1 and channel gating in Arabidopsis Plant Cell 2010 22 3076 3092 10.1105/tpc.110.077768 20884800 PMC2965544

[B7] Horak J Grefen C Berendzen KW Hahn A Stierhof YD Stadelhofer B The Arabidopsis thaliana response regulator ARR22 is a putative AHP phospho-histidine phosphatase expressed in the chalaza of developing seeds BMC Plant Biol 2008 8 77 10.1186/1471-2229-8-77 18625081 PMC2478664

[B8] Rashtchian A Thornton CG Heidecker G A novel method for site-directed mutagenesis using PCR and uracil DNA glycosylase PCR Methods Appl 1992 2 124 130 10.1101/gr.2.2.124 1477668

[B9] Little JW Mount DW Creating new restriction sites by silent changes in coding sequences Gene 1984 32 67 73 10.1016/0378-1119(84)90033-7 6099315

[B10] Zhang BZ Zhang X An XP Ran DL Zhou YS Lu J An easy-to-use site-directed mutagenesis method with a designed restriction site for convenient and reliable mutant screening J Zhejiang Univ Sci B 2009 10 479 482 10.1631/jzus.B0820367 19489114 PMC2689561

[B11] Zheng L Baumann U Reymond JL An efficient one-step site-directed and site-saturation mutagenesis protocol Nucleic Acids Res 2004 32 e115 10.1093/nar/gnh110 15304544 PMC514394

[B12] Liu H Naismith JH An efficient one-step site-directed deletion, insertion, single and multiple-site plasmid mutagenesis protocol BMC Biotechnol 2008 8 91 10.1186/1472-6750-8-91 19055817 PMC2629768

[B13] Qi D Scholthof KB A one-step PCR-based method for rapid and efficient site-directed fragment deletion, insertion, and substitution mutagenesis J Virol Methods 2008 149 85 90 10.1016/j.jviromet.2008.01.002 18314204

[B14] Park J Labaer J Recombinational cloning Curr Protoc Mol Biol 2006 Chapter 3 Unit 3.20 18265386 10.1002/0471142727.mb0320s74

[B15] Shankarappa B Vijayananda K Ehrlich GD SILMUT: a computer program for the identification of regions suitable for silent mutagenesis to introduce restriction enzyme recognition sequences Biotechniques 1992 12 882 884 1322684

[B16] Turchin A Lawler JF Jr The primer generator: a program that facilitates the selection of oligonucleotides for site-directed mutagenesis Biotechniques 1999 26 672 676 10343904 10.2144/99264st02

[B17] Breslauer KJ Frank R Blocker H Marky LA Predicting DNA duplex stability from the base sequence Proc Natl Acad Sci USA 1986 83 3746 3750 10.1073/pnas.83.11.3746 3459152 PMC323600

[B18] Freier SM Kierzek R Jaeger JA Sugimoto N Caruthers MH Neilson T Improved free-energy parameters for predictions of RNA duplex stability Proc Natl Acad Sci USA 1986 83 9373 9377 10.1073/pnas.83.24.9373 2432595 PMC387140

[B19] Sentenac H Bonneaud N Minet M Lacroute F Salmon JM Gaymard F Cloning and expression in yeast of a plant potassium ion transport system Science 1992 256 663 665 10.1126/science.1585180 1585180

[B20] Dreyer I Blatt MR What makes a gate? The ins and outs of Kv-like K+ channels in plants Trends Plant Sci 2009 14 383 390 10.1016/j.tplants.2009.04.001 19540150

[B21] Grefen C Obrdlik P Harter K The determination of protein-protein interactions by the mating-based split-ubiquitin system (mbSUS) Methods Mol Biol 2009 479 217 233 19083185 10.1007/978-1-59745-289-2_14

[B22] Tang W Ruknudin A Yang WP Shaw SY Knickerbocker A Kurtz S Functional expression of a vertebrate inwardly rectifying K+ channel in yeast Mol Biol Cell 1995 6 1231 1240 8534918 10.1091/mbc.6.9.1231 PMC301279

[B23] Grefen C Lalonde S Obrdlik P Split-ubiquitin system for identifying protein-protein interactions in membrane and full-length proteins Curr Protoc Neurosci 2007 Chapter 5 Unit 5.27 18428659 10.1002/0471142301.ns0527s41

